# Supplementary material for: Automated detection of the HER2 gene amplification status in Fluorescence in situ hybridization images for the diagnostics of cancer tissues
Source: Sci Rep. 2019 Jun 3;9:8231. doi: 10.1038/s41598-019-44643-z (PMC6546913; doi:10.1038/s41598-019-44643-z)
Supplement: Supplementary file 1 — Supplementary Information [file 41598_2019_44643_MOESM1_ESM.pdf]

Supplementary Information

for

Automated detection of the HER2 gene amplification status in Fluorescence *in situ* hybridization images for the diagnostics of cancer tissues

Falk Zakrzewski[1#], Walter de Back[2#], Martin Weigert[3,4], Torsten Wenke[5], Silke Zeugner[1], Robert Mantey[6], Christian Sperling[1], Katrin Friedrich[1+], Ingo Roeder[2,6], Daniela E. Aust[1,6], Gustavo Baretton[1,*] & Pia Hönscheid[1,6]*

<u>Institutional addresses</u>
1 Institute of Pathology, University Hospital Carl Gustav Carus (UKD), TU Dresden, Dresden, Germany
2 Institute for Medical Informatics and Biometry (IMB), Carl Gustav Carus Faculty of Medicine, TU Dresden, Dresden, Germany
3 Max Planck Institute of Molecular Cell Biology and Genetics (MPI-CBG), Dresden, Germany
4 Center for Systems Biology Dresden (CSBD), Dresden, Germany
5 ASGEN GmbH & Co. KG, Dresden, Germany
6 National Center for Tumor Diseases (NCT), Partner Site Dresden, Germany
***** *Corresponding author*
# Equal contribution
+ Deceased

Content

1. Supplementary Table S1

and

2. Supplementary Figure S1

Supplementary Table S1

for

Automated detection of the HER2 gene amplification status in Fluorescence *in situ* hybridization images for the diagnostics of cancer tissues

Falk Zakrzewski[1#], Walter de Back[2#], Martin Weigert[3,4], Torsten Wenke[5], Silke Zeugner[1], Robert Mantey[6], Christian Sperling[1], Katrin Friedrich[1+], Ingo Roeder[2,6], Daniela E. Aust[1,6], Gustavo Baretton[1,*] & Pia Hönscheid[1,6]*

Supplementary Table S1: Accuracies on 57 test images

num	acc_ND	acc_SD	image	k_ND	k_SD	Diff
0	0,5	0,560606061	1	0,344338566	0,445862209	-6,06%
1	0,777777778	0,777777778	2	-0,051921974	0,169724174	0,00%
2	0,833333333	0,8	3	0,7468863	0,789229484	3,33%
3	0,647058824	0,607843137	4	0,557003728	0,52677164	3,92%
4	0,833333333	0,833333333	5	0,8	0,857142857	0,00%
5	0,814814815	0,777777778	6	0,753895227	0,691083334	3,70%
6	0,733333333	0,666666667	7	0,435786436	-0,156545209	6,67%
7	0,714285714	0,595238095	8	0,61739063	0,497443163	11,90%
8	0,755555556	0,8	9	0,709569489	0,761266491	-4,44%
9	0,666666667	0,805555556	10	0,434640523	0,746732026	-13,89%
10	0,742424242	0,696969697	11	0,60375911	0,560949794	4,55%
11	0,771929825	0,824561404	12	0,544393312	0,729835736	-5,26%
12	0,623188406	0,623188406	13	0,454657133	0,525628785	0,00%
13	0,484848485	0,636363636	14	0,155803165	0,449338399	-15,15%
14	0,693333333	0,773333333	15	0,668047138	0,70946416	-8,00%
15	0,650793651	0,682539683	16	0,30861356	0,558241815	-3,17%
16	0,701149425	0,643678161	17	0,335087533	0,338181706	5,75%
17	0,62962963	0,722222222	18	0,158970097	0,51753588	-9,26%
18	0,538461538	0,628205128	19	0,225370451	0,245293344	-8,97%
19	0,535714286	0,666666667	20	0,395221661	0,364784314	-13,10%
20	0,793650794	0,777777778	21	0,367459738	0,273305814	1,59%
21	0,833333333	0,777777778	22	0,597979798	0	5,56%
22	0,666666667	0,747126437	23	0,554583564	0,520586	-8,05%
23	0,79047619	0,923809524	24	0,382971433	0,761582966	-13,33%
24	0,523809524	0,444444444	25	0,216082067	0,066853407	7,94%
25	0,655913978	0,698924731	26	0,375805967	0,371528267	-4,30%

26	0,666666667	0,753623188	27	0,456963905	0,593154523	-8,70%
27	0,6	0,706666667	28	0,346423232	0,45105632	-10,67%
28	0,558558559	0,522522523	29	0,217884766	0,222450704	3,60%
29	0,789473684	0,719298246	30	0,791306295	0,717511222	7,02%
30	0,75	0,694444444	31	0,830952381	0,61783986	5,56%
31	0,764705882	0,705882353	32	0,641177166	0,667801963	5,88%
32	0,871794872	0,948717949	33	0,779089235	0,940229885	-7,69%
33	0,623188406	0,710144928	34	0,410233144	0,616608139	-8,70%
34	0,966666667	0,966666667	35	0,968553459	0,968553459	0,00%
35	0,592592593	0,703703704	36	0,394766524	0,603351894	-11,11%
36	0,770833333	0,854166667	37	0,741837415	0,829059829	-8,33%
37	0,824561404	0,824561404	38	0,788078336	0,788078336	0,00%
38	0,770833333	0,8125	39	0,729286316	0,809900192	-4,17%
39	0,783333333	0,833333333	40	0,778639847	0,862102757	-5,00%
40	0,743589744	0,743589744	41	0,709936577	0,747956475	0,00%
41	0,80952381	0,880952381	42	0,716330876	0,846103391	-7,14%
42	0,777777778	0,844444444	43	0,495373271	0,743410853	-6,67%
43	0,666666667	0,777777778	44	0,32493828	0,485721908	-11,11%
44	0,739130435	0,68115942	45	0,429133848	0,488710087	5,80%
45	0,813333333	0,88	46	0,508696344	0,801708055	-6,67%
46	0,592592593	0,592592593	47	0,31690523	0,322222222	0,00%
47	0,65	0,666666667	48	0,515584984	0,542394425	-1,67%
48	0,6	0,722222222	49	0,375194217	0,339243445	-12,22%
49	0,666666667	0,766666667	50	0,501458665	0,557327035	-10,00%
50	0,675438596	0,763157895	51	0,37248348	0,485043048	-8,77%
51	0,666666667	0,696969697	52	0,465199348	0,623208512	-3,03%
52	0,756410256	0,653846154	53	0,348282779	0,298380454	10,26%
53	0,9	1	54	0,259259259	1	-10,00%
54	0,743589744	0,794871795	55	0,220169491	0,446224611	-5,13%
55	0,666666667	0,785714286	56	0,348648464	0,694645325	-11,90%
56	0,666666667	0,666666667	57	0,293574038	0,305755576	0,00%

<u>Supplementary Figure S1</u>

for

Automated detection of the HER2 gene amplification status in Fluorescence *in situ* hybridization images for the diagnostics of cancer tissues

Falk Zakrzewski[1#], Walter de Back[2#], Martin Weigert[3,4], Torsten Wenke[5], Silke Zeugner[1], Robert Mantey[6], Christian Sperling[1], Katrin Friedrich[1+], Ingo Roeder[2,6], Daniela E. Aust[1,6], Gustavo Baretton[1,*] & Pia Hönscheid[1,6]*

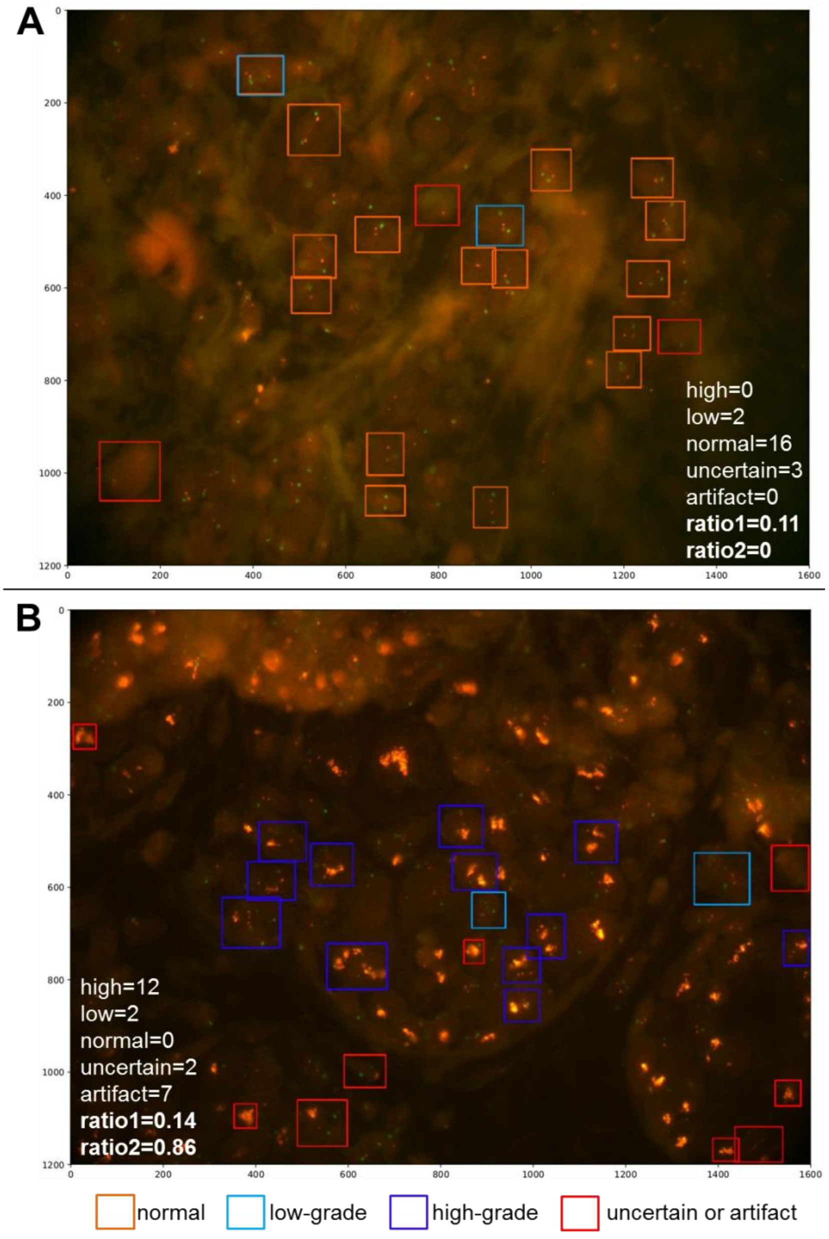

Supplemental Figure S1. Two examples of the application of our pipeline on FISH images of low quality.

In (**A**) a normal stage was detected and corresponds to the decision of a pathologist.

In (**B**) a high-grade stage was detected which also corresponds to the pathologists decision on the FISH image. Numerous nuclei have not been detected in both images indicating the limitations of our system on FISH images of very low quality. Training on a large set of FISH images of low quality would enhance the accuracy in detecting most nuclei.
